# Supplementary material for: Gene-specific RNA homeostasis revealed by perturbation of the NuA4/Tip60 acetyltransferase complex
Source: bioRxiv. 2024 Jan 31:2024.01.30.577960. Preprint. [Version 1] doi: 10.1101/2024.01.30.577960 (PMC10862879; doi:10.1101/2024.01.30.577960)
Supplement: Supplement 1 [file NIHPP2024.01.30.577960v1-supplement-1.pdf]

bioRxiv preprint doi: https://doi.org/10.1101/2024.01.30.577960; this version posted January 31, 2024. The copyright holder for this preprint (which was not certified by peer review) is the author/funder, who has granted bioRxiv a license to display the preprint in perpetuity. It is made available under aCC-BY 4.0 International license.

Supplementary Figures and Tables for

Transcript-specific RNA homeostasis revealed by perturbation of the NuA4/Tip60 acetyltransferase complex

By

Forouzanfar et al

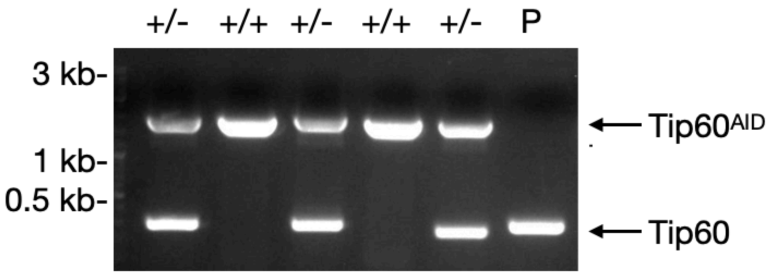

Supplementary Figure S1. Genomic PCR of independent mESC clones demonstrating the integration of the sequence AID-FLAG-BioTagP2A-EGFP into genomic loci of *Kat5* (Tip60) gene.

bioRxiv preprint doi: https://doi.org/10.1101/2024.01.30.577960; this version posted January 31, 2024. The copyright holder for this preprint (which was not certified by peer review) is the author/funder, who has granted bioRxiv a license to display the preprint in perpetuity. It is made available under aCC-BY 4.0 International license.

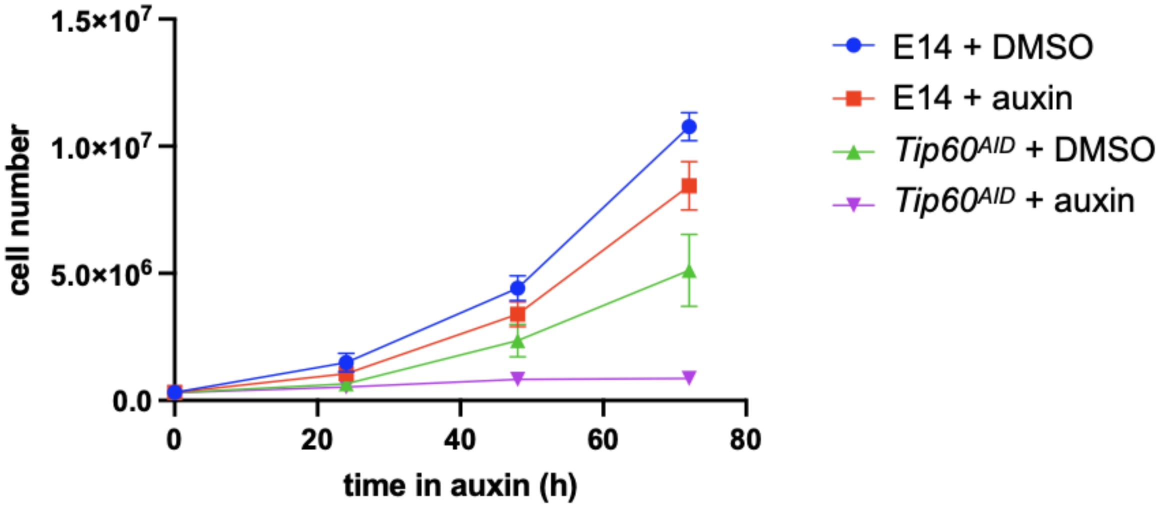

Supplementary Figure S2. Cell number (mean and SEM of n=3 independent experiments) of the indicated cells grown in LIF medium and treated with DMSO or 1 mM auxin.

bioRxiv preprint doi: https://doi.org/10.1101/2024.01.30.577960; this version posted January 31, 2024. The copyright holder for this preprint (which was not certified by peer review) is the author/funder, who has granted bioRxiv a license to display the preprint in perpetuity. It is made available under aCC-BY 4.0 International license.

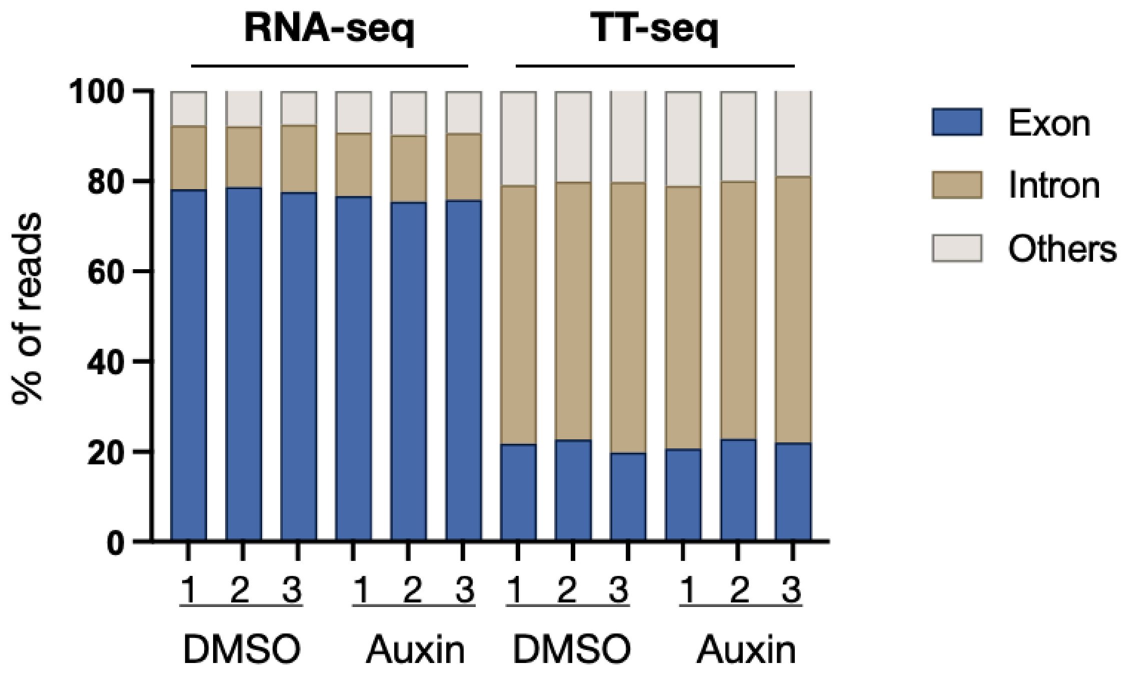

Supplementary Figure S3. Proportion of reads mapped to the indicated genomic elements for independent replicates (1-3) of RNA-seq and TT-seq experiments. Besides reads aligning against exons and introns, reads matching exon-intron junctions, exons-intergenic junctions and intergenic regions are represented as “others”.

bioRxiv preprint doi: https://doi.org/10.1101/2024.01.30.577960; this version posted January 31, 2024. The copyright holder for this preprint (which was not certified by peer review) is the author/funder, who has granted bioRxiv a license to display the preprint in perpetuity. It is made available under aCC-BY 4.0 International license.

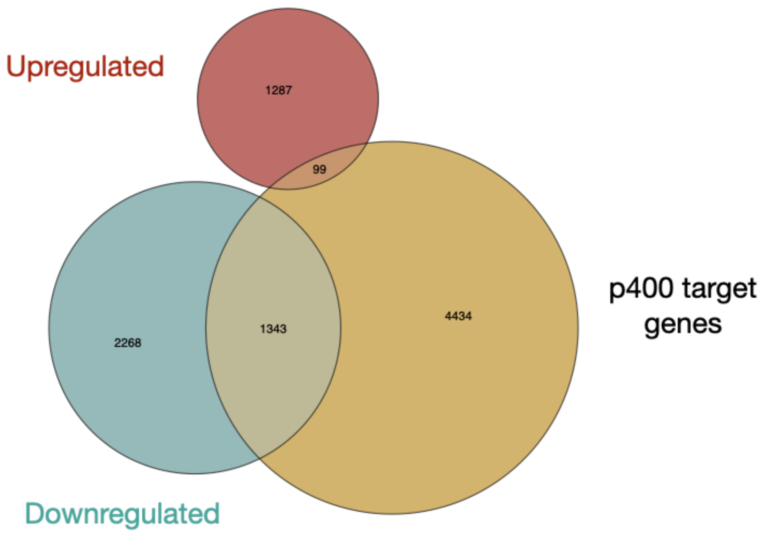

Supplementary Figure S4. Venn diagram designating the overlap between differentially expressed genes in DMSO vs auxin-treated *Tip60*^{AID} cells and p400-associated genes (Chen et al., 2015) assessed by TT-seq.

bioRxiv preprint doi: https://doi.org/10.1101/2024.01.30.577960; this version posted January 31, 2024. The copyright holder for this preprint (which was not certified by peer review) is the author/funder, who has granted bioRxiv a license to display the preprint in perpetuity. It is made available under aCC-BY 4.0 International license.

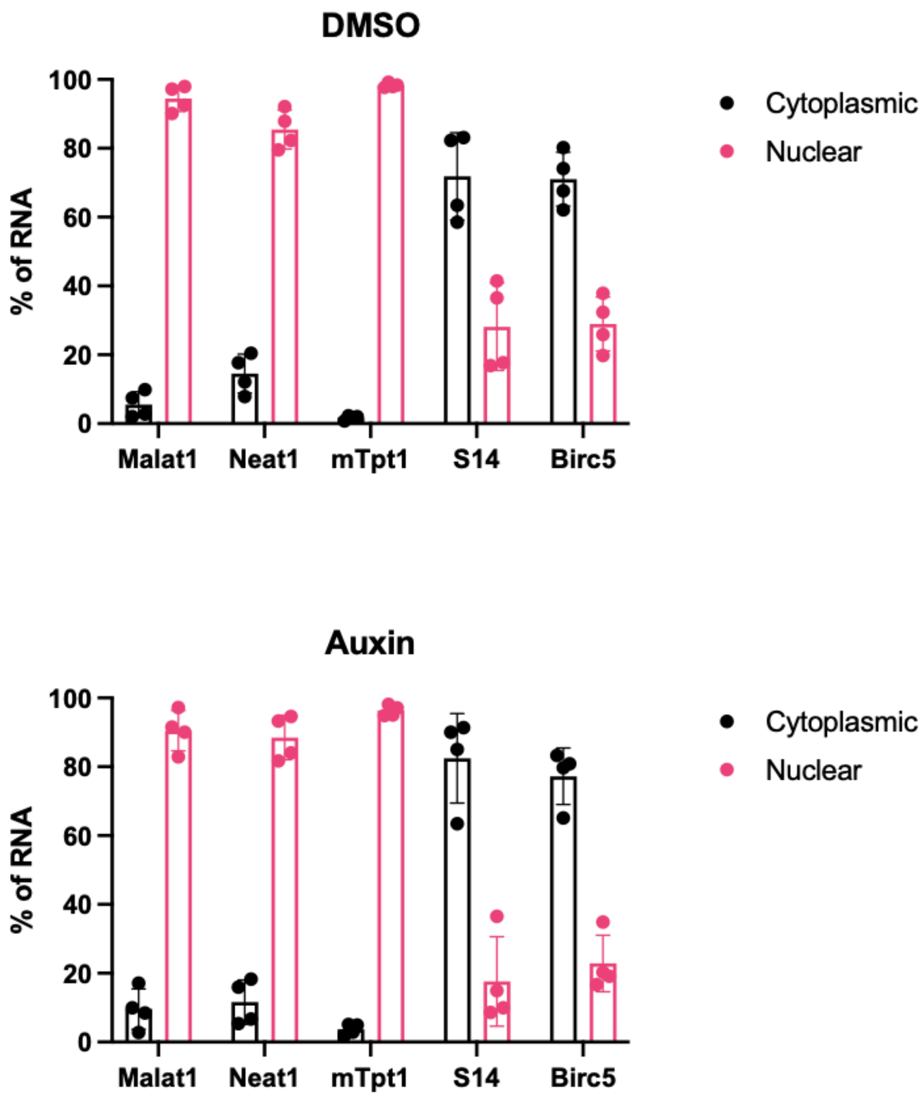

Supplementary Figure S5. RT-qPCR analysis of the indicated RNAs from nuclear and cytoplasmic fractions (mean and SD of n=4 biological replicates). Malat1 and Neat1 are nuclear lncRNAs. mTpt1 corresponds to an intronic region. Both RPS14 (S14) and BIRC5 mRNAs are expected to localise within the cytoplasm.

bioRxiv preprint doi: https://doi.org/10.1101/2024.01.30.577960; this version posted January 31, 2024. The copyright holder for this preprint (which was not certified by peer review) is the author/funder, who has granted bioRxiv a license to display the preprint in perpetuity. It is made available under aCC-BY 4.0 International license.

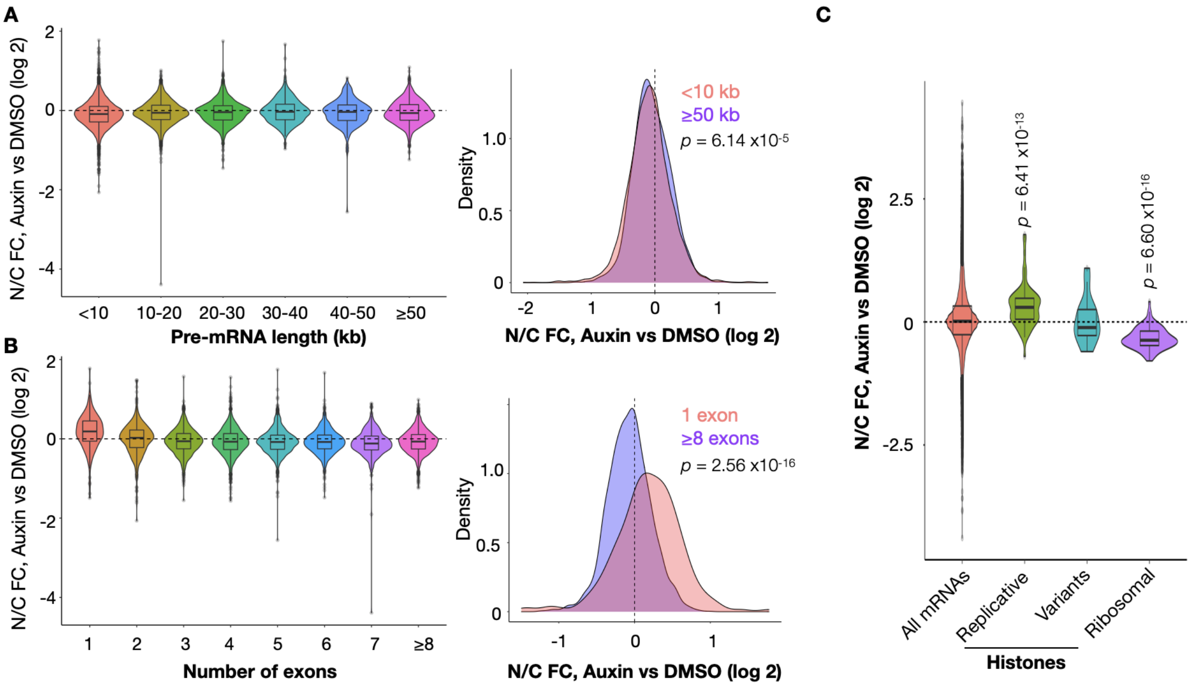

Supplementary Figure S6: TIP60 depletion leads to nuclear accumulation of intronless mRNAs. **A**: Violin plot of TIP60-dependent changes in nucleo/cytoplasmic ratio (auxin v DMSO fold change) for pre-mRNAs of varying lengths in 10 kb windows, and density plot showing that very short (<10 kb) and very long (>50 kb) pre-mRNAs are not significantly enriched in the nucleus after Tip60 depletion. **B**: Violin and density plots as in (A), but for pre-mRNAs containing various numbers of introns. Note that intronless pre-mRNAs show a slightly higher nuclear accumulation after TIP60 depletion. **C**: Violin plot as in (A-B) for specific mRNA classes. Note that replicative histone mRNAs are slightly enriched in the nucleus of TIP60-depleted cells, whereas ribosomal protein genes are slightly enriched in the cytoplasm.

bioRxiv preprint doi: https://doi.org/10.1101/2024.01.30.577960; this version posted January 31, 2024. The copyright holder for this preprint (which was not certified by peer review) is the author/funder, who has granted bioRxiv a license to display the preprint in perpetuity. It is made available under aCC-BY 4.0 International license.

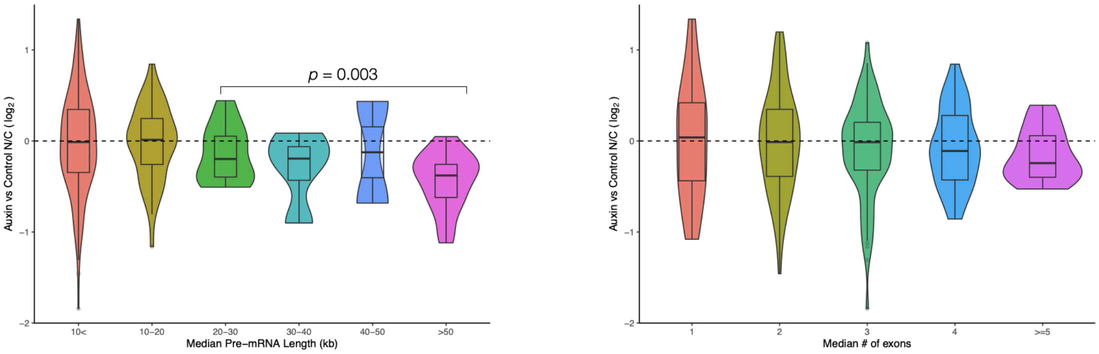

Supplementary Figure S7. Violin plot of TIP60-dependent changes in nucleo/cytoplasmic ratio (auxin v DMSO fold change) for lncRNAs of varying lengths in 10 kb windows (left) and for lncRNAs containing various numbers of introns. Note that lncRNAs of length >50 kb show a slightly higher cytoplasmic accumulation after TIP60 depletion.

bioRxiv preprint doi: https://doi.org/10.1101/2024.01.30.577960; this version posted January 31, 2024. The copyright holder for this preprint (which was not certified by peer review) is the author/funder, who has granted bioRxiv a license to display the preprint in perpetuity. It is made available under aCC-BY 4.0 International license.

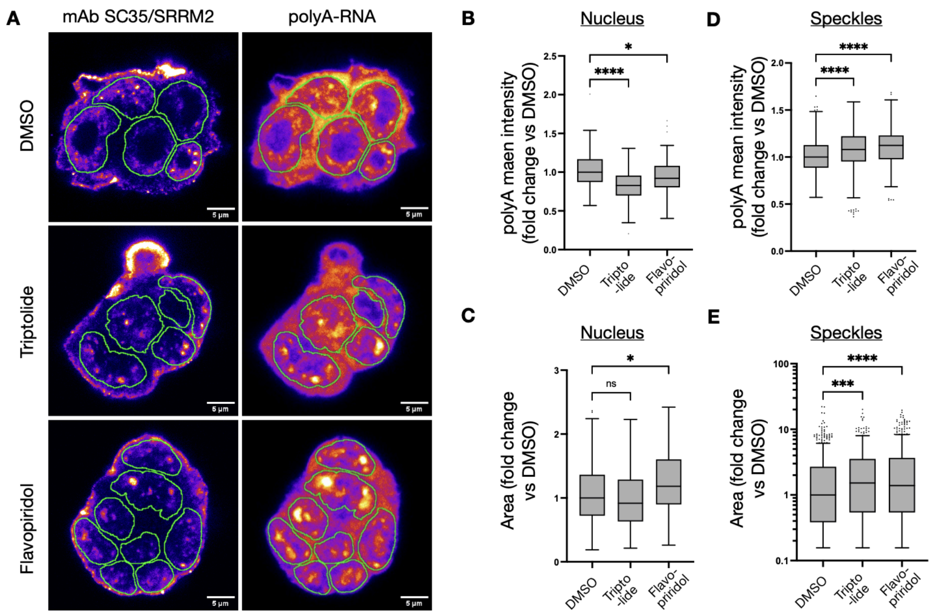

Supplementary Figure S8: Nuclear redistribution of mRNA upon inhibition of transcription. (**A**) Representative images of mouse embryonic stem cell colonies treated with DMSO, Triptolide, or Flavopiridol for 2 hours. Cells were fixed and stained with DAPI to label nuclei, SC35/SRRM2 monoclonal antibody labelling nuclear speckles, and Cy3-tagged polyT oligonucleotide labelling mRNA. Nuclear contours are depicted in green. Scale bar, 5 µm. (**B-E**) Box plots showing the fold change compared to the DMSO condition for the following parameters: (**B**) nuclear polyA mean intensity, (**C**) nuclear area, (**D**) polyA mean intensity on speckles, and (E) speckle area (note the logarithmic scale on the y-axis). Whiskers were calculated using the Tukey method. ns, not significant ($p > 0.05$); * $p \leq 0.05$; ** $p \leq 0.01$; *** $p \leq 0.001$; **** $p \leq 0.0001$. Cells were pooled from two independent experiments.

bioRxiv preprint doi: https://doi.org/10.1101/2024.01.30.577960; this version posted January 31, 2024. The copyright holder for this preprint (which was not certified by peer review) is the author/funder, who has granted bioRxiv a license to display the preprint in perpetuity. It is made available under aCC-BY 4.0 International license.

Supplementary Table 1. RT- qPCR primers used

Gene	Forward primer [5` => 3`]	Reverse primer [5` => 3`]
MALAT1	GGCCAGCTGCAAACATTCAA	TGCAGTGTGCCAATGTTTCG
NEAT1	GTACTGGTGAAGGTGTGGGG	TGTCGAGACAAGTATGCCCG
RPS14	GGAAACCATCTGCCGAGTGA	GTTTGATGTGCAGGGCAGTG
BIRC5	CGCGATTTGAATCCTGCGTT	AGGGCCAGTTCTTGAAGGTG
mTpt1	TTAAGCACATCCTTGCTAATTTCA	TGTACGAGACAGCAAACAGACTTT

4sU RNA-seq validation

Gene	Forward primer [5` => 3`]	Reverse primer [5` => 3`]
mTpt1	TTAAGCACATCCTTGCTAATTTCA	TGTACGAGACAGCAAACAGACTTT
mCfl1	TATGAGACCAAGGAGAGCAAGAA	GTTAAGCTCTGAGAAAGGGAACC

D. melanogaster spike

dRpl12	AAGGGAACCTGCAAGGAAGT	CCCTCGTTCAGTTCGTCAATA
Rp49	GACGCTTCAAGGGACAGTATCTG	AAACGCGGTTCTGCATGAG
